# P-2319. A 5-year Single Center Review of *Strongyloides stercoralis* Seropositivity in Cardiac Transplant Candidates in Central Texas

**DOI:** 10.1093/ofid/ofae631.2471

**Published:** 2025-01-29

**Authors:** Collin M Telchik, Andrea Grimbergen, Juan Negron-Diaz, Thao Giang, Jessica Lovelace, Audrene Edwards, Hector E Ramirez

**Affiliations:** Baylor Scott & White Medical Center, Temple, Texas; Baylor Scott & White - Temple, Temple, Texas; Baylor Scott & White Medical Center, Temple, Texas; Baylor Scott and White Medical Center- Temple, Temple, Texas; Baylor Scott and White Medical Center- Temple, Temple, Texas; Baylor Scott and White Medical Center- Temple, Temple, Texas; Baylor Scott & White Hospital, Temple, Texas

## Abstract

**Background:**

*Strongyloides stercoralis* is a parasitic nematode that chronically infects an estimated 30 - 100 million individuals worldwide and is mainly found in tropical, subtropical, and warm temperate regions. In solid organ transplant recipients, *Strongyloides* can cause disseminated disease or hyperinfection, which has an extremely high mortality rate. According to the American Society of Transplantation, all potential solid organ donors and recipients with risk factors for *Strongyloides* infection should undergo serologic screening. Our study aimed to identify the prevalence of *Strongyloides* seropositivity in patients evaluated for cardiac transplantation. We also aimed to identify demographic characteristics and risk factors associated with seropositivity, and any side effects of treatment.

Table 1

**Figure:**
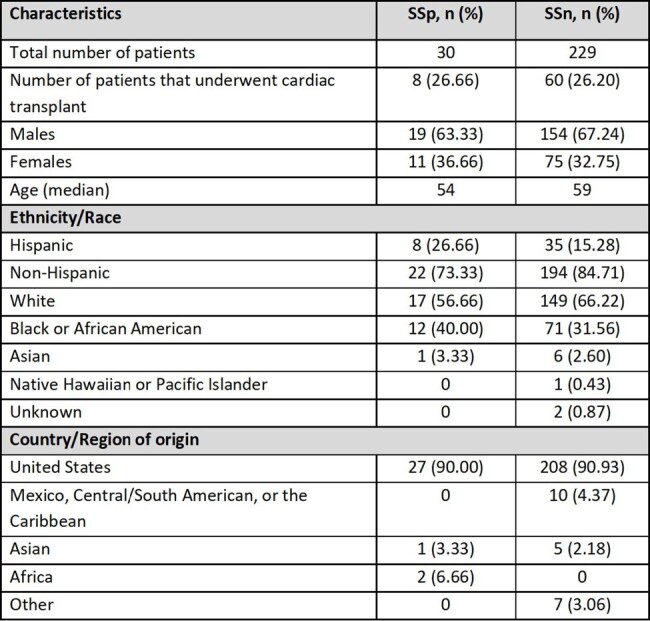

Total number of Strongyloides seropositive (SSp) and seronegative (SSn), total number of patients that received cardiac transplant, demographic characteristics, and country of origin.

**Methods:**

This study was a single-center, retrospective chart review of patients aged 18 or older who underwent cardiac transplant evaluation and serologic screening for *Strongyloides* at Baylor Scott & White Medical Center in Temple, Texas, between March 2019 and March 2024. A positive test was defined as > 1.1 Index Value (IV), negative as < 1.0 IV, and equivocal as 1.0-1.1 IV. Data was collected and managed using Research Electronic Data Capture (REDCap). Bivariate analyses were completed to test the association between the Strongyloides Seropositive (SSp) and Strongyloides Seronegative (SSn) groups. All statistical analyses were performed in SAS 9.4.

Table 2

**Figure:**
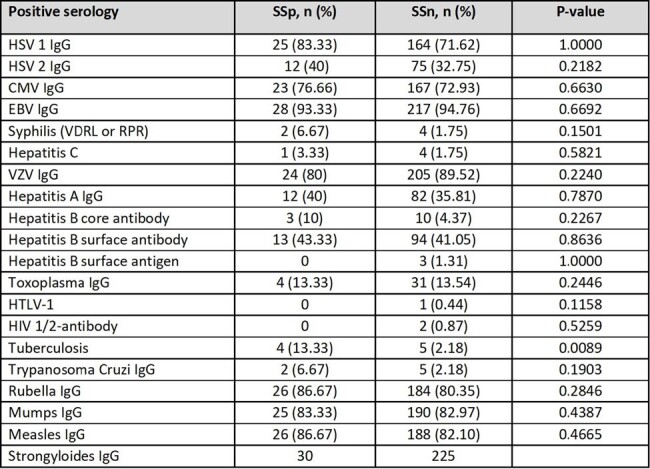

Pre-transplantation serologic results SSp and SSn. Abbreviations: Herpes Simplex Virus (HSV), Cytomegalovirus (CMV), Ebstein Bar virus (EBV), Varicella Zoster Virus (VZV), Human T-lymphotropic virus-1 (HTLV-1), human immunodeficiency virus (HIV).

**Results:**

259 patients underwent screening for cardiac transplant and *Strongyloides*. 11.58% tested positive or equivocal for *Strongyloides*. Epidemiological risk factors were not associated with seropositivity. All patients who underwent cardiac transplantation received appropriate treatment with no side effects. There were no cases of disseminated infection.

Table 3

**Figure:**
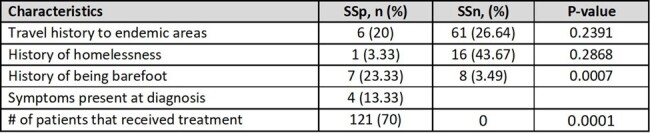

Travel history to endemic including tropic regions of Southeast Asia and the Appalachian region in the US, history of homelessness, history being barefoot, symptoms at diagnosis (Gastrointestinal, cutaneous, or respiratory symptoms), and treatment history.

**Conclusion:**

Our study provides insight into the prevalence of *Strongylodies* in Texas. Based on our results, targeted screening may miss many seropositive patients. Given the lack of epidemiological risk factors within our SSp population and rising concern for indigenous cases within Texas, we recommend consideration of universal *Strongyloides* screening for solid organ transplant donors and recipients.

**Disclosures:**

All Authors: No reported disclosures

